# Effects of verbatim repetition of the headline message on the proceed button on click-through rates in online retail

**DOI:** 10.3389/fpsyg.2024.1187798

**Published:** 2024-08-22

**Authors:** Florian Kutzner, Florian K. G. Ermark, Julian Fornoff, Michaela Wänke

**Affiliations:** ^1^Department for Economic and Consumer Psychology, Seeburg Castle University, Seekirchen am Wallersee, Austria; ^2^Institute of Psychology, Heidelberg University, Heidelberg, Germany; ^3^Department of Consumer and Economic Psychology, School of Social Sciences, University of Mannheim, Mannheim, Germany

**Keywords:** click-through rate, online shopping, processing fluency, conceptual fluency, repetition, field experiment

## Abstract

For online retailers, increasing click-through rates and reducing dropout rates are critical to success. In this study, we examine the effect of verbatim repetition of the website's headline message on the proceed button, based on research on processing fluency. In our field study involving 956 online platform visitors, we found that verbatim repetitions of the header message on the proceed button resulted in an increase in the conversion rate by more than 10 percentage points compared to gist repetitions and new messages. Our findings highlight the importance of simple verbatim features and demonstrate the successful application of processing fluency research to impact consumer behavior.

## Introduction

During the last few years, consumers have increasingly turned to online shopping (Bell et al., 2020). In 2016, for the first time, online purchases exceeded the number of purchases made in stores in the U.S. (Stevens, 2016). Since then, e-commerce has continued to expand globally and is projected to account for over 40% of worldwide retail sales by 2027 (BCG Global, 2023). In addition to goods, the online sales of on-demand services constitute an increasing market that brings new challenges (Xu et al., 2021). Not surprisingly, getting people to land on the websites of online shops has become a crucial goal for marketers, with two-thirds of the total advertising spending expected to go into digital advertising in the U.S. in 2023 (Haggin, 2023).

Getting potential customers to visit one's website is only a first step. Equally important, if not more so, is getting them to proceed to the offers, i.e., to click through. Thus, the so-called click-through rate (the proportion of visitors who actually click on a link or button) has become an important measure in consumer research (Robinson et al., 2007; Yang and Zhai, 2022). Although marketers invest a substantial amount of effort into optimizing conversion and click-through rates (King, 2008; Atkinson et al., 2014; Kumar and Salo, 2018), some estimates suggest that customers do not proceed to the end in more than 50% of all online transactions (Bell et al., 2020).

In response to this challenge, research focused on optimizing customer experience and boosting click-through rates. Most of this research focuses on usability and user experience, emphasizing user interface design elements such as graphics, colors, positioning, and font styles (Kim and Lennon, 2008; Im et al., 2010; Shneiderman et al., 2016; Nahai, 2017; Kumar and Salo, 2018). From a fluency perspective, the ease with which the graphic elements are processed creates a positive effect and facilitates action (Schwarz, 2004). Indeed, various studies in this field indicate the positive effects of experienced fluency in the processing of websites, producing a more positive shopping experience and increased purchase rates (Novak et al., 2003; Im et al., 2010; Mai et al., 2014; Mosteller et al., 2014).

The experience of fluency is, however, not limited to perceptual processing but also includes *conceptual fluency*, which refers to the ease with which the content can be comprehended and integrated into existing knowledge (Whittlesea, 1993). In the present study, we focus on conceptual fluency and how it may boost click-through behavior. More concretely, we examine some of the most basic elements of a website: headers that present a key message and buttons that allow customers to proceed with their purchase. We propose that matching the message conveyed in the header with the message conveyed on the proceed button will boost click-through rates.

### The impact of fluent processing

Processing fluency refers to the metacognitive experience with which new information is processed (Whittlesea, 1993; Schwarz, 2004; Alter and Oppenheimer, 2009). There are different dimensions of processing fluency: perceptual fluency processes lower-level physical attributes and features of stimuli, whereas conceptual fluency identifies a stimulus's meaning and its semantic associations (Whittlesea, 1993; Alter and Oppenheimer, 2009). Perceptual fluency is the ease with which people process sensory information of a stimulus, including physical qualities such as colors, forms, sizes, and orientations. The most prominent example of perceptual fluency effects is the mere exposure effect according to which repeated exposure increases the liking of the stimulus (Zajonc, 1968) because the facilitated processing produces positive affect which is attributed to the stimulus (Winkielman and Cacioppo, 2001). For example, in the context of consumer behavior, advertisements, brand names, logos, and products were evaluated better when participants had been exposed to them before (e.g., Janiszewski, 1988, 1993; Perfect and Askew, 1994; Lee and Labroo, 2004). In addition to repetition, easy-to-read font improves product evaluations (e.g. Gmuer et al., 2015), and high figure-ground contrast improve aesthetic judgments (Vogel et al., 2020). With regard to e-commerce, Lindgaard et al. (2006) found that an attractive, well-designed website could promote effective and fluid sensory processing.

Conversely, conceptual fluency refers to cognitive processing and includes comprehending, interpreting, and attaching meaning to the information given (Whittlesea, 1993; Lee and Labroo, 2004). The most prominent example of conceptual fluency effects is the illusory truth effect according to which repeated statements are considered to be more likely to be true than new statements (Hasher et al., 1977; Dechêne et al., 2009) presumably because repetition facilitates comprehension. Another factor that facilitates comprehension is the context in which a stimulus is encountered. For example, when products are shown in an environment typical for their use (e.g., ketchup in a hamburger restaurant), they are evaluated better compared to exposure in a less typical environment (Lee and Labroo, 2004). In one rare study examining conceptual fluency and product websites (van Rompay et al., 2010), product evaluations were better when the text and pictures matched rather than mismatched. Presumably, this is because the match made it easier to form an impression about the product. The familiarity of the customer with the material, their background knowledge, and the complexity of the language employed are among other things that may impact conceptual fluency.

Clearly, perceptual and conceptual fluency can additively affect consumer behavior and the overall shopping experience. Real consumer stimuli likely expose consumers to different sources of fluency at the same time (Whittlesea and Leboe, 2003). Thus, an e-commerce site with recognizable icons, an intuitive layout that encourages perceptual fluency, and clear, coherent content that encourages conceptual fluency could significantly improve the overall user experience and influence consumer engagement and purchase decisions.

Importantly, the impact of fluency is not limited to affecting product evaluations. Conceptual and perceptual fluency have been shown to influence consumption-related actions in multiple ways. For example, experiencing fluency while encoding instructions on how to proceed increased subjective ease and made proceeding more likely (Song and Schwarz, 2008; Mosteller et al., 2014). In the context of the present research experiencing fluency during the presentation of a persuasive message elicited more persuasion (Lee and Aaker, 2004; Lee et al., 2010; Vaughn et al., 2010) because presumably the experienced fluency creates “a feeling right” sensation, which encourages individuals to accept the message and align it with their initial thoughts regarding the message (Cesario et al., 2004; Lee and Higgins, 2009). In the same way, recent research indicates that processing fluency amplifies existing attitudes and action tendencies (Landwehr and Eckmann, 2020)^1^.

It is particularly the latter, namely, action tendencies that we address in the present research. We propose that when the content of the header and button on an online shopping site match, the action of clicking on the button will be facilitated. When the content represented on the button matches the content activated in the header, consumers will experience fluency, and this will reinforce the persuasiveness of the message in the header and the tendency to proceed with an action.

It is important to note that this hypothesis directly contradicts the marketing intuition that presenting multiple arguments and benefits is the best way to persuade consumers (e.g., “A traditional marketer would try to persuade people with more arguments”) (Sher and Lee, 2009; Sela and Berger, 2012; Zhao, 2018, p. 419; see also Ismagilova et al., 2019). However, the research on processing fluency suggests that less is often more when it comes to facilitating existing consumer behavior tendencies. Maximizing fluency by presenting the same benefit in the header and button of a website facilitates fluent information processing and reduces conflict. This reasoning is consistent with the research on consumer confusion (Walsh et al., 2007), which has shown that presenting less information typically leads to more positive evaluations, especially among customers who are not willing to engage in effortful information processing (Kang and Kim, 2006). Therefore, marketers may benefit from simplifying their messaging and focusing on presenting a single, clear benefit to consumers.

### Purpose of the present study

Based on these theoretical considerations, the present study tested how to label proceed buttons most effectively in accordance with the message of a website's header to create maximal fluency. More specifically, fluency should induce positive affect and a feeling of rightness. In addition, given that customers likely already have a latent interest in the offered service, fluency should amplify this pre-existing behavioral tendency (Landwehr and Eckmann, 2020). A high experience of fluency should consequently lead to higher click-through rates compared to no fluency experience.

As mentioned before, a marketer might want to serve multiple needs and thus add a novel message in the button label that complements the already presented message of the header. Adding another message could, however, result in relatively low fluency by increasing processing demands. Therefore, we suggest that appealing to the same themes in the button label that were already presented in the central message in the header is a more effective approach because it will induce higher fluency.

**Hypothesis 1**. Repetition of the messages activated in the header results in a higher click-through rate than presenting a new message in the button.

Still, the question remains how closely the message must be repeated. To merely avoid confusion and reduce cognitive processing demand, repeating the message in gist should suffice.

However, there is reason to believe that verbatim repetitions may have an advantage as a verbatim repetition of the header or parts of it would involve the least cognitive demand and facilitate processing without further effort. Particularly, consumers who only superficially scan the website and do not go into elaborate processing may be more affected by a verbatim repetition than a gist repetition.

**Hypothesis 2**. Verbatim repetition of the message presented in the header results in a higher click-through rate than a gist repetition of the presented message.

## Materials and methods

The study was conducted on three landing pages of the website of a German broker for home cleaning services. Visitors were randomly assigned to different conditions and automatically directed to the respective landing page. If a person visited the website multiple times during the period of the experiment, only the first visit was included. Since this field study used an existing website of a cooperation partner, no additional individual information including demographic data was obtained. No specific information regarding the page visit (i.e., time, place, or technical equipment) was collected to guarantee anonymity.

### Participants

An a priori power analysis using G^*^Power (Faul et al., 2007) for the planned logistic regression indicated a required sample size of 797 for a targeted power of 0.8, aiming for an effective increase of the click-through rate of at least 5% when assuming the default click-through rate on the landing page to be in the midrange (OR = 1.22). These odds were derived based on the findings that the dropout rate in online shopping is assumed to be at least 50% or more (Bell et al., 2020). We used 50% as the benchmark because an increase from 50% to 55% click-through rate allows for the most conservative criterion to demonstrate the hypothesized effect with the intended power. Using a cooperation partner's real website posed a constraint, as we could not easily switch between the experimentally manipulated and the original landing pages to reach exactly the calculated sample size. Thus, based on previous visitor numbers, we estimated that a period of 3 weeks should suffice to attain the minimum required sample size. This resulted in a total of 956 visitors that were included in the analyses, thereby warranting adequate power.

### Design

Since the company had already experimented with different button labels, there was no pre-existing baseline button. Therefore, we introduced a design that combines the used messages in the button with the ascribed experimental conditions to ensure that potential effects were not due to one particular wording. The experiment followed a 2 (header) x 2 (button label: new vs. repetition) x 2 (nested factor of repetition: gist vs. verbatim) factorial research design. The eight conditions are shown in Table 1.

**Table 1:** Overview of the combinations of header claims and button labels for all eight conditions.

**Header**	**Dreaming**				**No Stress**			

	**“Dreaming instead of Cleaning Get there faster: With XXX your cleaning help is found quickly and easily”**				**“No stress with cleaning Book your cleaner now with XXX without searching for long”**			
**Buttons**	**Message repetition in button**		**New message in button**		**New message in button**		**Message repetition in button**	
	**Verbatim repetition**	**Gist repetition**					**Verbatim repetition**	**Gist repetition**
	“At last, time for dreaming”	“Relax now”	“A clean apartment without any stress”	“No more cleaning duties”	“At last, time for dreaming”	“Relax now”	“A clean apartment without any stress”	“No more cleaning duties”

Translation of the original German headers and buttons.

We used two headers of different content (“Dreaming” and “No stress”) to exclude that effects are due to a particular phrasing of the message. The header message was either repeated on the button or a different message was presented on the button (message repetition vs. new message). For the nested factor in the case of message repetition, the wording either repeated parts of the header verbatim or used a different wording addressing the same theme (gist vs. verbatim repetition). Thus, out of the eight conditions, two represented a verbatim message repetition (one for each header), and two represented a gist repetition (one for each header). The remaining four conditions resulted from combining each header with both the verbatim and gist repetition of the other header. In these conditions, the messages in the button addressed different aspects than those in the header.

### Materials

**Figure 1:**
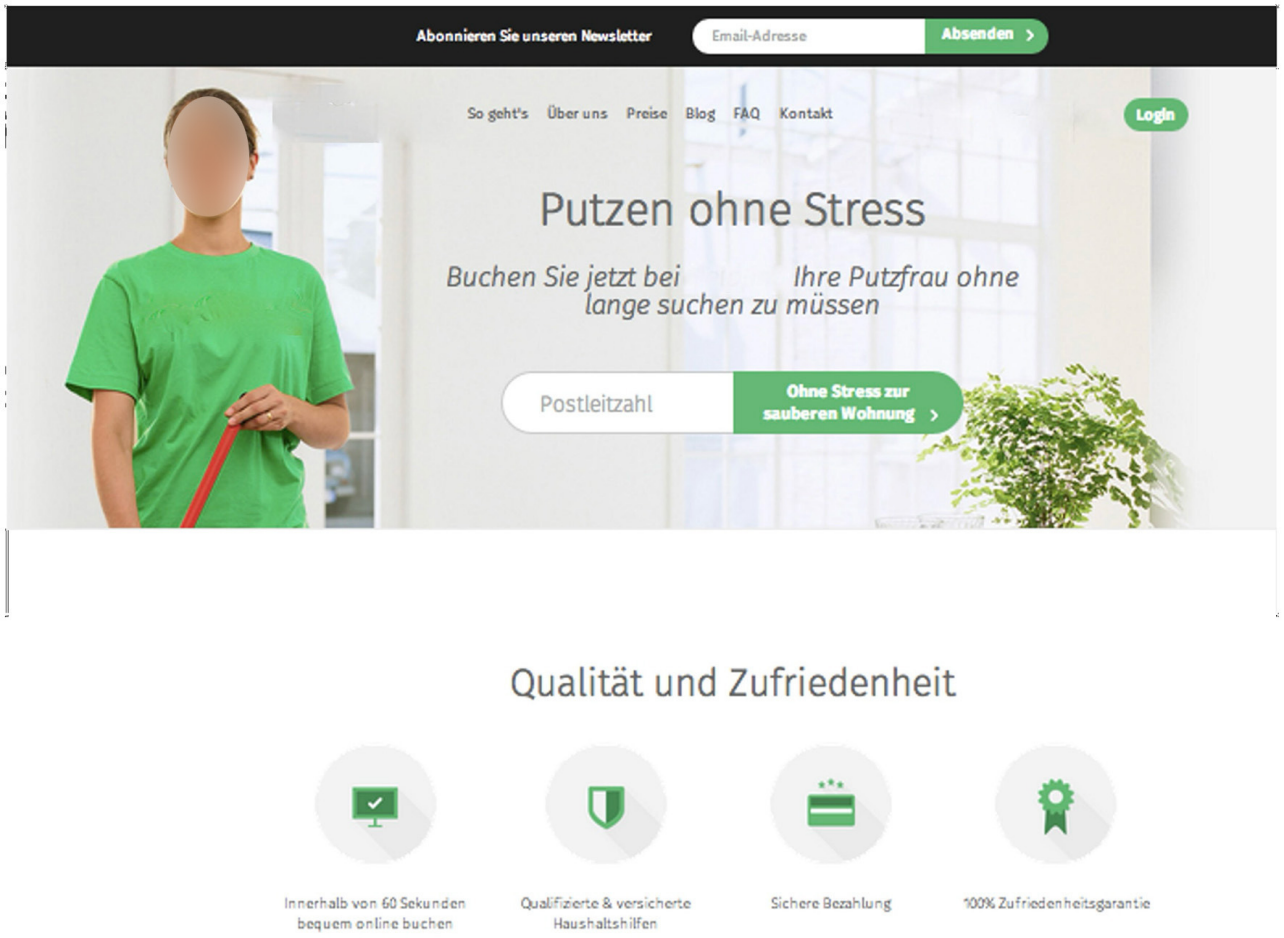
An example of the verbatim repetition landing page using the “stress” theme, titled “Ohne Stress zur sauberen Wohnung” (without stress to a clean home).

We created eight different landing pages by combining one of the two headers with the respective button that either represented a verbatim or gist repetition, or with one of the two buttons addressing a new message. Both the header and the button were clearly visible in the landing page's upper section. The button was positioned to the right of a text field where visitors had to enter their zip code to access the available offers. The background of the upper section featured an image of a cleaner wearing a shirt with the company's logo, standing in a brightly lit room (c.f. Figure 1).

The “Dreaming” header activated the theme of having time for dreaming instead of cleaning. The corresponding button in the verbatim repetition condition repeated the word “dream”. The gist repetition referred to relaxation as thematically similar without mentioning the word “dream”. In contrast, the “No stress” header associated the service with stress reduction. Here, the verbatim repetition directly repeated the word “stress”, whereas the gist repetition used cleaning duties as a thematically similar stimulus. Since the two headers address different themes, the buttons corresponding verbatim or in gist to one header served new message buttons for the other header. In other words, each button text represented a repetition of the header's message (either verbatim or in gist) in one (header) condition but a new message in the other (header) condition. This crossed usage of the four different messages in the button labels is a particular strength as each message is once used as a repetition and a new message.

### Statistical procedure

The dependent variable, click-through rate, was calculated by dividing the number of people clicking on the button by the total number of page visitors. Therefore, we tracked the number of visitors and their clicking behavior. For every visitor, click-through was either rated as “yes”(1) or “no”(0).

To analyze click-through rates, we calculated a logistic regression to test the influence of the different button labels. To compare the different conditions, we implemented the Helmert contrasts. The first contrast compared the new message conditions with the message repetition conditions. The second contrast compared the gist repetition with the verbatim repetition conditions. To test for potential differences of those effects depending on the type of header, we also included a centered variable indicating the type of header and, critically, its interactions with the contrast variables. All analyses were conducted using R (R Core Team, 2021) and RStudio (Posit team, 2022). The two different header claims were coded as binary (1 = “Dreaming” header, 0 = “No stress” header). The match of the messages in the button texts with the header claims was coded as either verbatim (message) repetition, gist repetition, or new message.

## Results

The average descriptive click-through rate across all conditions was 48.22%. The click-through rate was highest for the two conditions with verbatim message repetition (*N* = 237), *M* =0.58, followed by the two conditions with gist message repetition (*N* = 234), *M* = 0.48. The four conditions with a new message presented in the button showed the lowest click-through rate (*N* = 485), *M* = 0.44 (c.f. Table 2 for descriptive values of each condition by header theme).

**Table 2:** Descriptive statistics for the conversion rate of each condition (new message, gist repetition, and verbatim repetition) by header theme.

**Header**	**Dreaming**			**No Stress**		
**Buttons**	**Message repetition in button**		**New message in button**	**New message in button**	**Message repetition in button**	
	**Verbatim repetition**	**Gist repetition**			**Verbatim repetition**	**Gist repetition**
	M = 0.58, SD = 0.50, *N* = 126	M = 0.51, SD = 0.50, *N* = 112	M = 0.42, SD = 0.49, *N* = 245	M = 0.45, SD = 0.50, *N* = 240	M = 0.59, SD = 0.49, *N* = 111	M = 0.45, SD = 0.50, *N* = 122

**Figure 2:**
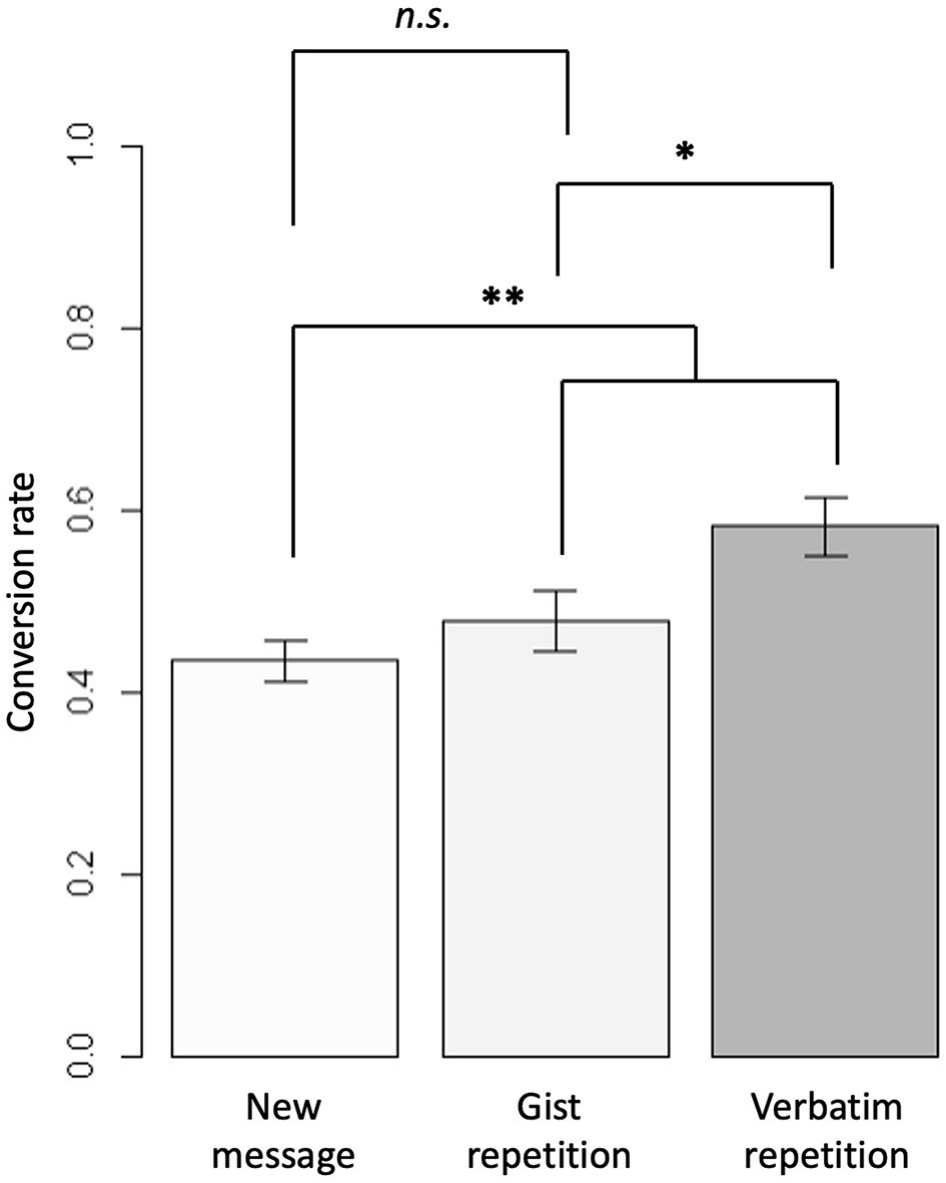
Average conversion rates (i.e. click-through rates) for different button conditions and illustration of the statistical comparisons. Lower brackets represent comparison with Helmert contrast for first, ^**^p = 0.003, and second contrast, ^*^p = 0.024. The upper bracket represents an exploratory comparison of gist repetition with the presentation of a new message in the button text, p=0.307. Error bars indicate standard errors of the mean.

The logistic regression analysis, χ(2) = 15.10, *p* < 0.001, *Nagelkerke R*^2^ = 0.021, with the Helmert contrasts resulted in a significant effect for the first contrast, *b* = 0.13 (SE = 0.04), *z* = 2.97, *p* = 0.003, *OR* = 1.14, with a 95% CI [1.05, 1.24], comparing the click-through rate of the new message conditions with the message repetition conditions, as well as for the second contrast, *b* = 0.21 (SE = 0.09), *z* = 2.22, *p* =0.027, *OR* = 1.23, with a 95% CI [1.02, 1.48], comparing the gist repetition with the verbatim repetition conditions (see the brackets in Figure 2 for the comparisons and their respective significance levels). Neither the effect of the header type nor its interaction with the Helmert contrasts reached the conventional levels of significance, *z*'s < 1, *p*'s > 0.39.

The exploratory analysis revealed that the click-through rate did not differ significantly between presenting a new message on the button and a gist repetition, χ^2^(1) = 1.042, *p* = 0.307, *OR* = 0.84, with a 95%CI [0.61, 1.16] (compare Figure 2, upper bracket, for descriptive values), indicating an overall dominance of the verbatim repetition.

Furthermore, to determine whether the observed effect was due to the more appealing button labels in the verbatim repetition conditions, we directly compared the two button labels of the verbatim repetition conditions and those same labels when paired with a non-matching header in the new message conditions. The results confirm that the effects observed cannot be attributed to the wording of the buttons *per se* (e.g., a stronger message of some buttons) but must be related to the match between the button and the header, χ^2^(1) = 5.932, *p* = 0.015, *OR* = 1.59, with a 95%CI [1.09, 2.32]. This highlights the relevance of a match between the message presented in the header and the button label, emphasizing even further the importance of a direct match between the two texts in terms of phrasing.

## Discussion

We addressed the question of how to label buttons most effectively to increase the click-through rate on websites. The present study showed that a verbatim match between a message presented in the header of a website and the wording on the button increases the click-through rate. The addition of a new claim in the button label to provide multiple arguments in favor of the service was much less successful.

This is consistent with the findings that presenting stimuli that fit a previously activated concept is easier to process and create a fluent experience (Lee and Labroo, 2004; Schwarz, 2004). However, according to this literature, a button presenting a thematically similar label rather than a direct, verbatim repetition should also create positive affect through the experience of fluency, thereby increasing the click-through rate. In our study, the gist repetition of the header message in other wording fell behind the verbatim repetition. We suggest that this phenomenon is due to the reduced processing effort associated with direct, verbatim repetition that likely creates the strongest fluency experience akin to mere-exposure effects (Winkielman et al., 2003; Alter and Oppenheimer, 2009; Dechêne et al., 2009).

### Limitations and future research

It was beyond the limitations of the current field experiment to identify which of the multiple effects of increased fluency (e.g., higher credibility, increased persuasion, experienced effort, or amplified pre-existing behavioral tendencies) increased the click-through rate. However, it should be noted that the contribution of these effects is not mutually exclusive. It is possible that different reasons contributed to proceed with the action.

While we predicted the advantage of verbatim repetition, we also hypothesized that the gist repetition with a similar message in the button would have an advantage over a new message. This difference was not significant. Possibly what have been designed as similar messages were not similar enough. Perhaps, the terms “dreaming” and “relaxing” on one side and “no stress” and “no duties” on the other did not activate the same or close enough concepts, respectively. The relatively limited space on a button also restricts the length and detail of the message it can convey. This limitation makes it more difficult for a short phrase that does not repeat verbatim the original claim to trigger the same concept. Thus, we cannot dismiss the possibility that other, more similar concepts in the header and the button would prove successful (e.g., dreams and fantasies or stress and strain).

Since the study was conducted on a cooperation partner's real website, we could not acquire additional information about the customers to test mediating processes such as perceived ease of processing, difficulty in understanding, perceived truth, or time to process (Kostyk et al., 2021). Furthermore, the website used in this study offered only one specific type of service. However, the overall theoretical framework and prior research suggest that there is no reason to assume the findings should not be generalizable across other types of service providers or retail settings.

Future research should address further process variables in different environments to investigate the different impacts of fluency. Investigating the demonstrated effect in an enriched online shopping environment with multiple products is an important next step to examine whether the introduced technique could help boost one product over other competing products or brands.

### Conclusion and managerial implications

Simple text elements, such as the verbatim repetition of messages in a website's header and on the presented proceed buttons, may have a more substantial impact than online marketers are aware of. Creating a fluent shopping experience by inducing a feeling of ease when processing repeated messages on buttons might be a valuable marketing strategy to keep customers from quitting online shopping early, counteracting significant customer dropout rates. Supporting the customer's pre-existing behavioral tendencies or attitudes can create more involved customers with higher obstacles to leave the page or cancel a booking procedure.

The study demonstrates that, in addition to extensive investments in visual design features and user experience analyses, a straightforward tool to increase the click-through rate on the landing page is to focus on simple text features and their respective labeling. This method involves low investment costs and has the potential for strong effects, yielding a meaningful increase in sales returns. Thus, applying insights from fluency research and addressing the combined effects of conceptual and perceptual aspects of fluency could prove to be economically more profitable than expensive superficial design features.

## Data availability statement

The raw data supporting the conclusions of this article will be made available by the authors, without undue reservation.

## Ethics statement

Ethical approval was not required for the studies involving humans because the research was part of a master's thesis at the University of Mannheim for which no compulsory ethical approval exists. No deception was involved. Participants were not identifiable and no personal data was collected by the researchers. The studies were conducted in accordance with the local legislation and institutional requirements. Written informed consent for participation was not required from the participants or the participants' legal guardians/next of kin in accordance with the national legislation and institutional requirements because the research was part of the ongoing web-interface testing of a commercial website under the German GDPR regulation.

## Author contributions

FE and FK were engaged equally in the preparation of the manuscript. JF was involved in the design, the implementation of the experiment, and provided a first draft of the manuscript. MW gave repeated input for the theoretical arguments and edited the manuscript. All authors contributed to the article and approved the submitted version.

## Footnotes

^1^Fluency can also have a negative effect if negative constructs are activated beforehand or when negative attitudes or behavioral tendencies exist (Landwehr and Eckmann, 2020).
